# Impacts of different types of straw returning on soil physicochemical properties, microbial community structure, and pepper quality

**DOI:** 10.3389/fpls.2025.1620502

**Published:** 2025-07-01

**Authors:** Xiapu Gai, Biao Chen, Shijuan Xiong, Qingqing Zhou, Yuan Yuan, Yumei Mou

**Affiliations:** ^1^ Guizhou Key Laboratory of Molecular Breeding for Characteristic Horticultural Crops, Institute of Pepper, Guizhou Academy of Agricultural Sciences, Guiyang, China; ^2^ Anshun College of Agriculture, Anshun, China

**Keywords:** straw returning, soil microbial community, pepper quality, karst areas, high-throughput sequencing technology

## Abstract

**Introduction:**

Straw returning serves as a critical agronomic practice for soil quality improvement and sustainable agricultural development. However, the differential regulatory mechanisms of cellulose-rich (e.g., pepper straw) versus lignin-rich (e.g., mulberry stem) straw types on soil physicochemical properties, microbial community structure, and crop nutritional quality remain poorly understood, particularly in pepper cultivation regions of Karst mountainous.

**Methods:**

In this study, taken the pepper planting soil as research object, the treatments of cellulose pepper straw (CS), lignin mulberry stem (MS) and control (CK) were set up. By measuring soil nutrients, microbial community diversity and pepper nutritional quality indicators, combined with high-throughput sequencing technology, the ecological effects of different types of straw returning were systematically analyzed.

**Results and discussion:**

The results showed that CS treatment significantly increased nitrate nitrogen content and available potassium, but decreased organic matter content by 8% and microbial biomass nitrogen by 65.5%. MS treatment significantly increased soil organic matter and microbial biomass carbon, and promoted the increase of amino acid content by 59.6%. Microbial community analysis showed that *Pseudomonas* had the highest values in all three treatments, CS treatment enriched *Actinomycetota*, while MS treatment inhibited *Acidobacteriota*. Correlation analysis revealed that the functions of bacteria and fungi were positively correlated with SOC, NO_3_^–^N, AP, TK, MBN, and EC, reaching a significant level with MBN (*P*<0.05). However, they showed negative correlation with NH_4_^+^-N. It indicated that straw types drive microbial functional differentiation by regulating nutrient availability. The results of chili quality showed that CS treatment significantly increased capsaicin (16.5%) and crude fiber (21.4%), but reduced reducing sugar (13.3%). MS treatment increased amino acids (59.6%) and crude fat (6.3%), which was related to the different metabolic pathways caused by the difference in carbon-nitrogen ratio. In summary, cellulose-based straw enhances nutrient availability through short-term mineralization, indicating that carbon pool loss and nitrogen imbalance risks might limit long-term soil health. However, lignin-based straw maintains long-term soil health through carbon sequestration and microbial homeostasis, which provides a theoretical basis for the utilization of straw resources in karst areas and the improvement of quality and efficiency of pepper industry.

## 1 Introduction

Chili pepper (*Capsicum annuum L.*) is an important economic crop and condiment raw material in China, and its industry occupies a strategic position in rural revitalization and agricultural sustainable development (Zou et al., 2020). Guizhou Province, as the core producing area of pepper in China, has a planting area of 5.86 million mu, with a total output of more than 8 million tons and an annual output value of more than 35 billion yuan in 2023. It was accounted for 15% of the total output of pepper in China (Guizhou Provincial Department of Agriculture and Rural Affairs, 2023). Pepper planting covers more than 1 million households in the province, with an average net income of about 2500 yuan per mu, which has become a pillar industry to consolidate the achievements of poverty alleviation. However, limited by the soil impoverishment, poor water and fertilizer conservation and microclimate heterogeneity in karst areas, the average yield of pepper in Guizhou is only 2.1 tons per mu, which is significantly lower than the national average (3.5 tons/mu) (Mou et al., 2020). Therefore, improving soil health and pepper quality has become the core proposition of high-quality development of pepper industry in Guizhou.

Straw returning is a key way to improve the quality of continuous cropping soil, which reshapes soil ecological function through carbon and nitrogen input and microbial regulation (Berhane et al., 2020; Huang et al., 2021a). Recent studies have shown that straw returning could improve the stability of soil organic carbon pool and promote nutrient cycling through priming effect (Jin et al., 2020a; Xin et al., 2024). For example, based on the 27-year long-term experiment of wheat-maize rotation in the North China Plain, the authors confirmed that the soil organic carbon pool of straw returning reached stability after 15 years of returning and the growth rate before saturation was 1.8%. Meanwhile, straw returning helped to enhance soil nitrogen retention capacity (Gai et al., 2019). A meta-analysis containing 1822 pairs of observations from 78 studies shoued that straw return increased grain yield and quality of three main crops (maize, rice, and wheat) (Zhang et al., 2024b). High cellulose straw (such as corn straw and pepper straw) promotes the formation of aggregates and the proliferation of bacteria actinomycetes through rapid decomposition, thereby enhancing the turnover of available nutrients (Cong et al., 2025; Qin, 2021). Meanwhile, high lignin straw (such as wheat straw) enhances the fungal network and humification process through the slow-release of phenolic substances, thereby improving carbon sequestration stability (Wang et al., 2023a; Huang et al., 2021b). Interestingly, in karst areas, returning straw to the field had improved soil structure to a certain extent, reduced the erosion, runoff and sediment on the surface of caused by rainfall, increased the number of soil microorganisms, and played a role in water and soil conservation (Wen et al., 2019). It is worth noting that straw types significantly affected the functional differentiation of soil microorganisms. Cellulose straws (such as chili straws) accelerated carbon mineralization by enriching degrading bacteria such as *Cellvibrio* and *Streptomyces* (Bhattacharjee et al., 2023). Lignin straws (such as mulberry) activated the phenylpropanoid metabolic pathway by recruiting bacteria such as *Pseudomonas* and *Sphingomonas* (Wang et al., 2023b). However, existing studies have focused on a single straw type, and there is still a lack of systematic comparison of the differences in soil improvement and microbial mechanisms between crop straws dominated by cellulose and lignin.

As the core driver of nutrient transformation, the composition and activity of soil microbial community were directly regulated by the chemical properties of straw. Studies have shown that *Pseudomonadota* and *Actinomycetota* played a key role in cellulose degradation and nitrification (Yuan et al., 2023b), while *Acidobacteriota* was sensitive to soil pH and phenolic substances, and its abundance might decrease due to the accumulation of lignin decomposition products (Alami et al., 2021). In addition, the dynamic changes of microbial biomass carbon (MBC) and nitrogen (MBN) could reflect the stability of soil active nutrient pool. High C to N ratio straws maintained the balance of microbial biomass nitrogen by prolonging the decomposition cycle, while low C to N ratio straws might cause nitrogen fixation or loss (Vance et al., 1987). These processes not only affected soil nutrient availability, but also might regulate the synthesis of secondary metabolites such as capsaicin, vitamin C and amino acids through the soil-microorganism-plant interaction network (Zou et al., 2022a). However, most of the existing studies focused on single straw type, and the synergistic response mechanism of soil microbial community and pepper quality under different straw returning modes is still unclear.

The author’s institution is engaged in scientific research on chili peppers and sericulture and mulberry. According to the statistics, Guizhou Province produced more than 3 million tons of pepper straw and 525,000 tons of mulberry branch resources per year (Guizhou Provincial Bureau of Statistics, 2023), but the utilization rate was less than 20%. Random disposal leads to soil compaction and non-point source pollution (Ye et al., 2020). So the authors conducted this two types of straw returning experiments by combining two industries. Scientific utilization of two types of straw resources was of great significance to realize the green and sustainable development of agriculture. However, the differential mechanisms of the two types of straw in soil carbon and nitrogen sequestration, microbial community structure and pepper quality regulation are not yet clear. To this end, this study used macrobase sequencing technology to study the pepper soil in Guizhou, and set up cellulose pepper straw (CS) and lignin mulberry straw (MS) returning treatment. By analyzing soil physical and chemical properties, microbial community structure and pepper quality indicators, the differentiation mechanism of different types of straw on soil improvement was clarified. This would provide a theoretical basis for improving the quality and efficiency of Guizhou pepper industry and recycling of agricultural waste.

## 2 Materials and methods

### 2.1 Testing site

The experimental site was located in the research base of pepper institute, Guizhou Academy of Agricultural Sciences (26°24′N, 106°32′E). The altitude of the experimental area was 1100 m, the rainfall was 1470 mm, and the annual average temperature was 15.0 °C. It belongs to the subtropical monsoon climate zone. The soil belongs to yellow soil. The basic physical and chemical properties of the soil were as follows: pH was 6.83, organic matter was 18.8 g kg^-1^, total nitrogen was 1.65 g kg^-1^, alkali-hydrolyzed nitrogen was 74.4 mg kg^-1^, available phosphorus was 9.2 mg kg^-1^, available potassium was 561 mg kg^-1^.

### 2.2 Testing materials

The raw materials used in the experiment were pepper straw and mulberry stem, labeled as CS (chili pepper straw) and MS (mulberry stem), respectively. The carbon content of pepper straw was 418.8 g kg^-1^, the nitrogen content was 17.2 g kg^-1^, and the carbon to nitrogen ratio was 24.3. The carbon content of mulberry stalk was 442.5 g kg^-1^, the nitrogen content was 11.3 g kg^-1^, and the carbon to nitrogen ratio was 39.2. The test pepper *(Capsicum annuum L.)* variety is Layan 101, which is a variety selected by the Pepper Institute of Guizhou Academy of Agricultural Sciences.

### 2.3 Experimental design

This study adopted a randomized block design and implemented straw returning during land preparation in early April 2024. The test straw (CS and MS) was crushed by a cutting machine to a particle size of ≤ 2 cm. According to the treatment requirements, the application rates of 100 kg/667 m² (equivalent to 1.5 t/ha) and 500 kg/667 m² (7.5 t/ha) were evenly spread on the surface, and immediately mixed with soil at a depth of 20 cm using a rotary tiller. The treatment of not applying straw to the field as a control. Each treatment was repeated three times with a plot area of 120 m^2^ (15m × 8m). Nitrogen fertilizer was urea, phosphorus fertilizer was superphosphate, potassium fertilizer was potassium sulfate, the application amount is N 350 kg hm^-2^, P_2_O_5_ 129 kg hm^-2^, K_2_O 439 kg hm^-2^, organic fertilizer application amount is 600 kg hm^-2^, 70% nitrogen fertilizer, all phosphorus fertilizer, potassium fertilizer and organic fertilizer are used as base fertilizer, which are applied before the planting of pepper. 30% nitrogen fertilizer as topdressing.

In early March, the floating seedling technology was used to raise seedlings. It was planted in early May. The ditch was 130 cm, the width of the box was 80 cm, the width of the ditch was 50 cm and the height of the ridge was 20 cm. 6 - 8 leaves of seedling age with relatively consistent growth were selected to start transplanting. One plant per hole. Pepper was harvested in mid-August. The planting density was 2565 plants per 667 m^2^. The plant spacing was 40 cm and the row spacing was 50 cm. In addition to the different types of returning fields, other field management measures were carried out in accordance with the habits of local farmers and remained consistent.

### 2.4 Field sampling and experimental analysis

#### 2.4.1 Soil sample collection

After the pepper was harvested, the straw was removed from the field, and a soil auger with a diameter of 2.5 cm was used. Five 0-20 cm soil cores were randomly taken *in situ* in each plot with an “S”-shaped sampling route. After mixing evenly, impurities such as plant residual roots and gravel were removed for testing. Some samples were used to determine soil pH, organic carbon, soil total nitrogen, total phosphorus, total potassium, ammonium nitrogen, nitrate nitrogen, available phosphorus, available potassium, conductivity, cation exchange capacity and other related indicators after different treatments. Some samples were immediately frozen in liquid nitrogen and stored in the laboratory at -80°C refrigerator for soil microbial biomass and microbial community structure analysis.

#### 2.4.2 Pepper sample collection

Ten pepper plants with consistent growth were selected from different treatments with basically the same field management level. Random sampling method was used to take fresh commercial fruits with no pests and diseases above the second layer of pepper, uniform size and full red ripeness. Some of them were measured for water content, and some were dried to constant weight by hot air at 80°C. After removing the fruit stalk and crushing, they were passed through a 60-mesh sieve for the determination of total carbon, total nitrogen, capsaicin, vitamin C, crude protein, total amino acid content, total sugar content, reducing sugar, crude fat, crude fiber, dry matter and other nutritional quality indicators of pepper fruit.

#### 2.4.3 Measuring methods

Soil pH and nutrients were all analyzed using conventional methods (Lu, 2000). Soil pH was measured with deionized water (1:5 soil/water). Content of soil organic matter was analyzed with dichromate oxidation, and content of soil total nitrogen by the Kjeldahl digestion method (KDY-9830, Beijing). Content of soil total phosphorus was determined using NaOH melting-molybdenum antimony colorimetric method and content of soil total potassium using NaOH melting-flame photometry method. Soil ammonium nitrogen (NH_4_^+^-N) and nitrate nitrogen (NO_3_^–^N) concentrations were determined colorimetrically with a Flow Injector Auto Analyzer (Auto Analyzer 3, High Resolution Digital Colorimeter) after extracting 12 g of soil with 100 mL 0.01 M CaCl_2_. The analytical methods were described in details by Gai et al. (2016). Electrical conductivity (EC) was measured by conductivity meter. Cation exchange capacity (CEC) was determined by ammonium acetate exchange method-ammonium nitrogen colorimetric method.

Soil microbial biomass C (MBC) and microbial biomass N (MBN) were analyzed with the chloroform fumigation-extraction method (Vance et al., 1987). A value of 0.45 was used for the fraction of mineralized biomass C (K_C_) and 0.68 for the fraction of mineralized biomass N (K_N_). Specifically, 10 g (dry weight equivalent) of the soil that was stored at 4 °C was placed in a 100 mL glass beaker and fumigated in the dark for 24 h with alcohol-free chloroform. One soil beaker without fumigation was set up as a control. Both fumigated and unfumigated samples were extracted with 40 mL of 0.5 M K_2_SO_4_ solution by shaking in a thermostatic shaker at 25°C and 200 rpm for 30 min, followed by filtering (soil:water = 1:4). The concentrations of C and N in the extracts were determined by an Automated TOC/TN Analyzer (Multi N/C, 3100/HT1300, Analytik Jean, Germany). Dissolve organic C (DOC) and dissolved organic N (DON) were calculated by using the unfumigated soil samples.

Analysis of soil microbial community structure using metagenomic sequencing technology (Rastogi and Sani, 2011). Firstly, genomic DNA was extracted from 0.5 g soil samples using Mobio Power Soil DNA Isolation Kit, and DNA quality was detected by 1% agarose gel electrophoresis and Nanodrop. Subsequently, the V3-V4 region of bacterial 16S rRNA (primer 338F/806R) and the V1 region of fungal ITS (primer 1737F/2043R) were amplified by PCR, and the MiSeq double-end sequencing was completed by Shanghai Meiji Biomedical Technology Co., Ltd. The original sequence was subjected to QIIME2 v2019.7 quality control processing, including denoising, splicing and chimera removal, to obtain high-quality sequences (clean tags). The Uparse software was used to cluster into operational taxonomic units (OTUs) at 97% similarity, and the RDP classifier algorithm was used to compare the SILVA and UNITE databases for species annotation. Venn and Circos plots showed OTU distribution, and PCoA analysis evaluated community β diversity based on Bray-Curtis distance matrix. LEfSe analysis screened differential species (LDA > 3.0), and Pearson correlation heat map and Mantel Test revealed the association between environmental factors and flora. Functional prediction was performed by PICRUSt against the KEGG database.

Determination of nutritional quality in pepper fruits. Determination of total carbon, total nitrogen, and capsaicin content were quantified using high-performance liquid chromatography (HPLC) according to Chinese National Standard GB/T 21266-2007 “*Determination of Capsaicinoids in Chili and Its Products and Expression of Pungency Intensity*” (Shim et al., 2013); Vitamin C, crude protein, and total amino acids were analyzed via ninhydrin colorimetric method (Subroto et al., 2020); Total sugar and reducing sugar were determined following GB 5009.7-2016 “*Determination of Reducing Sugards in Foods*”*;* Crude fat content was measured using a crude fat analyzer (GB 5009.6-2016 “*Determination of Fat in Foods*”; Crude fiber content was assessed via filter bag technique (GB/T 5009.10-2003 “*Determination of Crude Fiber in Plant-Based Foods*”). Dry matter content was determined by the drying method.

### 2.5 Data analysis

Data were analyzed using SPSS 22.0, with analysis of variance (ANOVA) employed to evaluate the effects of different types of straw returning on soil nutrient content and microbial biomass. Figures were generated using Origin 9.0.

## 3 Results

### 3.1 Effects of different treatments on soil nutrients and microbial biomass contents

The response characteristics of soil nutrients to different straw return treatments showed significant differences (**Table 1**). Among the treatments, CS increased soil pH by 0.1 units, while MS decreased soil pH by 0.3 units, though the differences between treatments were not significant. Compared to CK, CS decreased SOM content by 8%, whereas MS increased SOM by 10%. Both CS and MS increased TN content by 11%, TP by 67%, and TK by 5.2% and 0.5%, respectively, relative to CK. For soil NH_4_^+^-N, CS and MS treatments reached levels 2.3-fold and 2-fold higher than CK. Notably, CS increased soil NO_3_^-^-N content to 3.1 times that of CK, while MS significantly reduced NO_3_^-^-N by 70.6%. Both straw return treatments enhanced AK content, CS and MS increased AK by 83% and 42.6%, respectively, relative to CK. For soil EC, CS resulted in a 3.5-fold higher EC than CK, whereas MS had no significant effect on EC. In terms of soil CEC, both CS and MS decreased CEC by 34.3% and 11.7%, respectively, compared to CK. Compared to CK, CS and MS treatments increased soil MBC by 9.3% and 20.4%, respectively. In contrast to the changes in MBC, MS enhanced soil MBN by 52.9% relative to CK, whereas CS reduced MBN by 65.5%.

**Table 1:** Statistical data of soil nutrients under different treatments.

Soil Nutrients	CK	CS	MS
pH	7.3 ± 0.1 ^a^	7.4 ± 0.3 ^a^	7.0 ± 0.2 ^b^
Soil organic matter (SOM) (g/kg)	13.8 ± 0.7 ^ab^	12.7 ± 0.5 ^b^	15.2 ± 1.3 ^a^
Total nitrogen(TN) (g/kg)	1.8 ± 0.1 ^b^	2.0 ± 0.2 ^a^	2.0 ± 0.1 ^a^
Total phosphorus(TP) (g/kg)	1.2 ± 0.1 ^b^	2.0 ± 0.2 ^a^	2.0 ± 0.2 ^a^
Total potassium(TK) (g/kg)	19.2 ± 0.2 ^a^	20.2 ± 0.1 ^a^	19.3 ± 0.7 ^a^
Ammonium nitrogen(NH_4_^+^-N) (mg/kg)	0.6 ± 0.1 ^c^	1.4 ± 0.3 ^a^	1.2 ± 0.2 ^b^
Nitrate nitrogen(NO_3_^-^-N) (mg/kg)	6.8 ± 1.7 ^b^	21.2 ± 3.2 ^a^	2.0 ± 0.2 ^c^
Available phosphorus(AP) (mg/kg)	53.2 ± 0.9 ^b^	85.5 ± 3.3 ^a^	55.5 ± 5.1 ^b^
Available potassium(AK) (mg/kg)	307.5 ± 23.9 ^c^	562.0 ± 85.1 ^a^	438.5 ± 27.3 ^b^
Electrical conductivity (EC) (μS/cm)	146.1 ± 10.7 ^b^	516.3 ± 72.5 ^a^	157.5 ± 22.0 ^b^
Cation exchange capacity (CEC) (cmol/kg)	23.0 ± 1.9 ^a^	15.1 ± 2.8 ^c^	20.3 ± 3.3 ^b^
Microbial biomass carbon(MBC) (mg/kg)	101.7 ± 14.2 ^b^	111.2 ± 20.0 ^ab^	122.4 ± 14.3 ^a^
Microbial biomass nitrogen(MBN) (mg/kg)	38.0 ± 4.1 ^b^	13.1 ± 2.6 ^c^	58.1 ± 4.1 ^a^

Different lowercase letters following the data in the same row indicate significant differences among treatments at the *P* < 0.05.

### 3.2 Effects of different treatments on soil microbial community structure

#### 3.2.1 Analysis of Venn diagram

**Figure 1:**
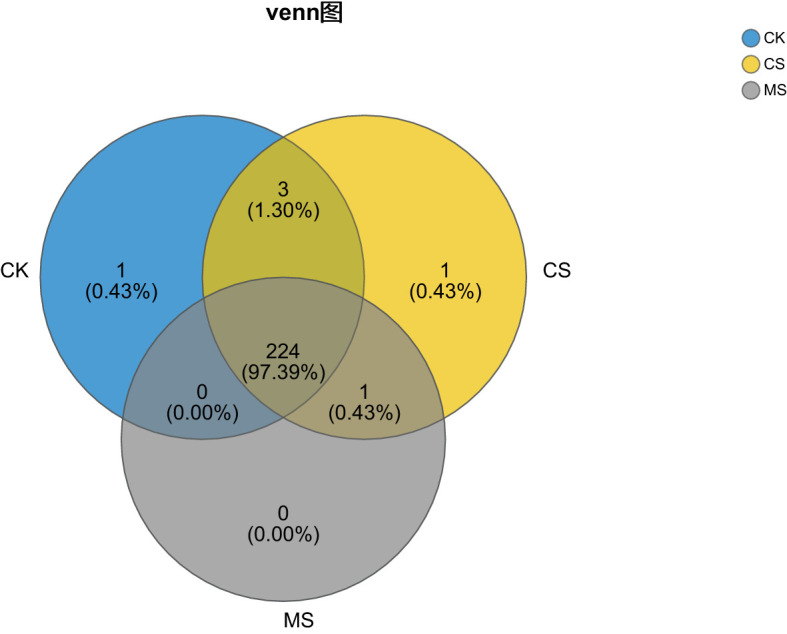
Venn diagram illustrating the species distribution at the phylum level in soil samples under different treatments.

The Venn diagram visually reflects the differences and overlaps in soil bacterial community OTUs composition among different treatments. In this study, a 97% sequence similarity threshold was used to define OTUs (**Figure 1**). The results showed that 224 OTUs were shared among the three treatments, accounting for 98.2%, 97.8%, and 99.6% of the total OTUs in CK, CS, and MS, respectively. Additionally, CK had 1 unique OTU (0.43% of total OTUs), CS had 1 unique OTU (0.43%), and MS had no unique OTUs. These data indicate that different treatments did not significantly affect the compositional differences in soil OTUs. Pepper straw return (CS) slightly increased the number of bacterial OTUs but did not lead to the formation of unique bacterial species. Furthermore, the type of straw (CS vs. MS) had no significant impact on this proportion.

#### 3.2.2 Bar chart of community distribution and circos diagram of species distribution

**Figure 2:**
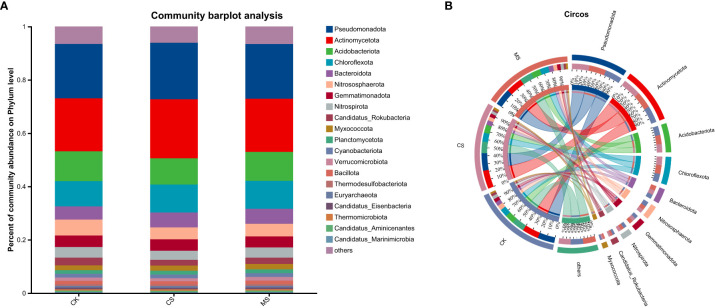
Bar chart **(A)** of community composition at the phylum level and circos diagram **(B)** of species co-occurrence networks in soil samples under different treatments.

Through high-throughput sequencing, soil samples from the three treatments were classified at the phylum level with a 97% similarity threshold, revealing a total of 231 bacterial and fungal phyla and 5,232 bacterial and fungal genera (including unclassified taxa and others) (**Figure 2**). Phyla with an average relative abundance greater than 1% were defined as dominant bacterial phyla, comprising 23 phyla that collectively accounted for over 90% of the total community. Among these, *Pseudomonadota* (formerly *Proteobacteria*) showed the highest relative abundance across all treatments (20.3%-20.6%). Other dominant phyla included *Actinomycetota* (19.8%-22.3%), *Acidobacterio* (9.9%-11.3%), and *Chloroflexota* (9.5%-10.6%).

The Circos diagram of sample-species relationships illustrates the distribution of microbial taxa across different soil treatments. At the phylum level, *Pseudomonadota* (formerly *Proteobacteria*) was the dominant phylum in all treatments. Its relative abundance showed no significant differences among CK, CS, and MS (20.3%-20.6%). In contrast, *Actinomycetota* increased from 19.8% in CK to 22.3% in CS, while *Acidobacteriota* decreased from 11.2% in CK to 10.0% in CS. No significant variation was observed in *Chloroflexota* abundance between CS and MS treatments.

#### 3.2.3 PCoA plot

**Figure 3:**
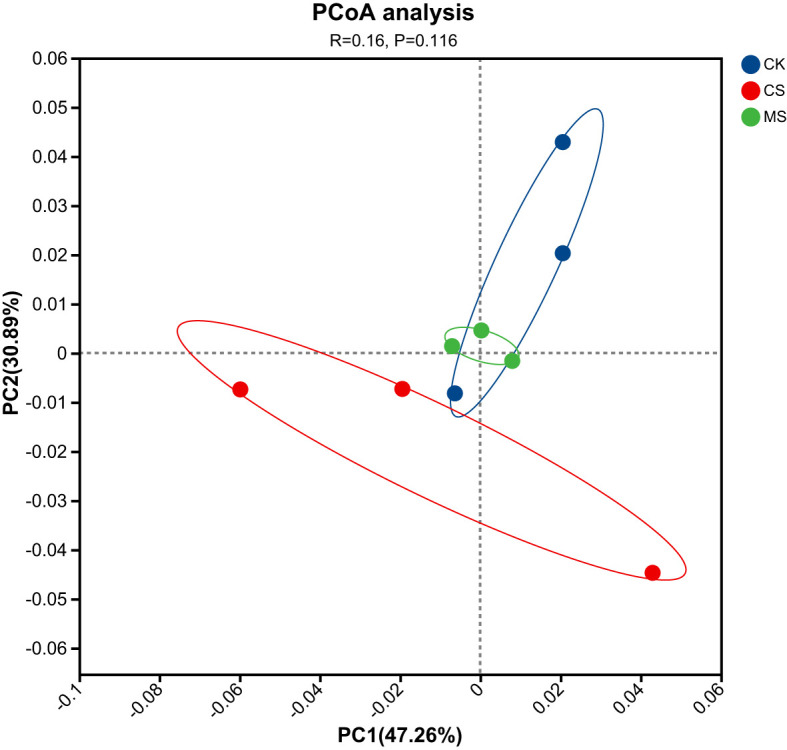
PCoA plot of microbial communities across different treatments.

Beta diversity analysis of microbial communities across treatments revealed distinct clustering patterns (**Figure 3**). The PCoA results demonstrated that Principal Coordinate 1 (PC1) and PC2 accounted for 47.26% and 30.89% of the variance, respectively. A significant separation was observed between the CS and CK treatments, while the MS and CK groups overlapped, indicating that pepper straw returning significantly altered soil microbial communities, whereas mulberry straw amendments had no statistically discernible impact.

#### 3.2.4 LEfSe-based hierarchical discriminant analysis of differentially abundant taxa

**Figure 4:**
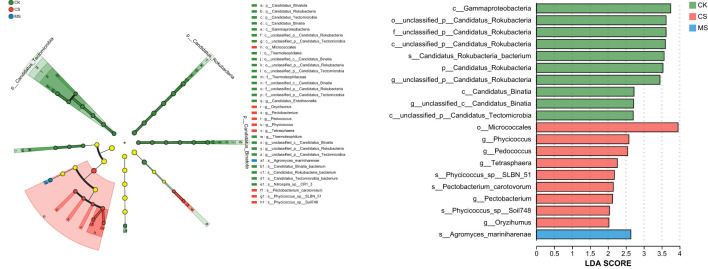
LEfSe-based multi-level discriminant analysis of differentially abundant taxa across treatments.

Linear discriminant analysis ffect Size (LEfSe) revealed that the relative abundances of signature fungal and archaeal microorganisms in soil varied with the application of different types of straw amendments. The results of the multi-level species discriminant analysis using LEfSe across different soil treatments are shown in **Figure 4**. A total of 34 bacterial and archaeal taxa exhibited significant differences. The CK treatment had the highest number of differential taxa, primarily belonging to the genus *Gammaproteobacteria*. In contrast, the mulberry straw treatment showed the fewest differential taxa (only one), identified as *mariniharenae* within the genus *Agromyces*. CS treatment contained nine differential taxa, with the highest-scoring taxon being *Micrococcales* at the genus level.

### 3.3 Analysis of soil nutrient effects on soil microbial community structure

**Figure 5:**
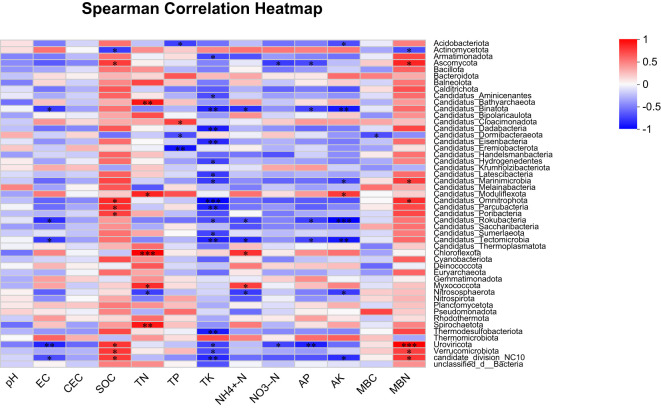
Heatmap analysis of soil nutrients and microbial community structure. * represents reaching a significant level at the p<0.05 level; ** represents reaching a significant level at the p<0.01 level; *** represents reaching a highly significant level at the p<0.01 level.

The correlation heatmap visually represents the relationships between microbial taxa/functional traits and environmental factors, highlighting both the magnitude and significance of their correlations. Pearson correlation analysis (**Figure 5**) revealed significant positive associations between SOM, TN, NH_4_^+^-N, AK, MBN and taxa such as *Ascomycota*, *Moduliflexota*, *Omnitrophota*, *Chloroflexota*, *Myxococcota*, *Uroviricota*, *Verrucomicrobiota*, and *NC10*. Conversely, TP, TK, NO_3_^-^-N, and AK were negatively correlated with *Rokubacteria*, *Binatota*, *Candidatus Tectomicrobia*, *Nitrososphaerota* and *Uroviricota*. Notably, pH showed no significant impact on microbial taxa abundance. These findings suggest that straw amendments alter microbial community structures primarily by modifying soil physicochemical properties, which varied depending on the straw type applied.

**Figure 6:**
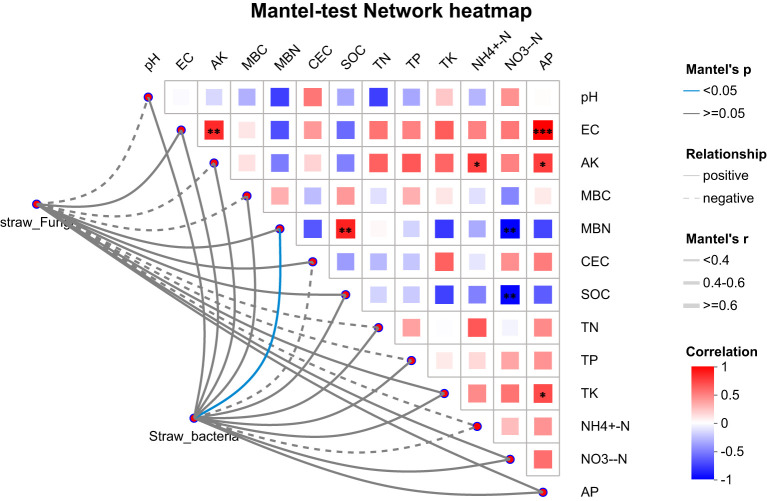
Mantel test analysis of soil nutrients and microbial community structure. * represents reaching a significant level at the p<0.05 level; ** represents reaching a significant level at the p<0.01 level; *** represents reaching a highly significant level at the p<0.01 level.

The Mantel test, combined with a network heatmap, is widely used in microbiome studies to assess correlations between environmental factors and microbial community structures. Mantel test revealed significant relationships between straw amendments and soil physicochemical properties (**Figure 6**). EC exhibited highly significant positive correlations with AP and AK. MBN showed a highly significant positive correlation with SOC. AK was significantly positively correlated with AP, TN, TK, and TP. Functional traits of both bacteria and fungi were positively associated with SOC, NO_3_^-^-N, AP, TK, MBN, and EC, with the correlation to MBN reaching statistical significance (*P* < 0.05). Conversely, these traits were negatively correlated with NH_4_^+^-N. Notably, pH, AK, MBC, TN, and TP displayed divergent relationships, showing that positive correlations with bacterial functions but negative correlations with fungal functions. CEC correlated positively with fungal functions but negatively with bacterial functions.

### 3.4 Effects of different treatments on the nutritional quality of pepper

**Figure 7:**
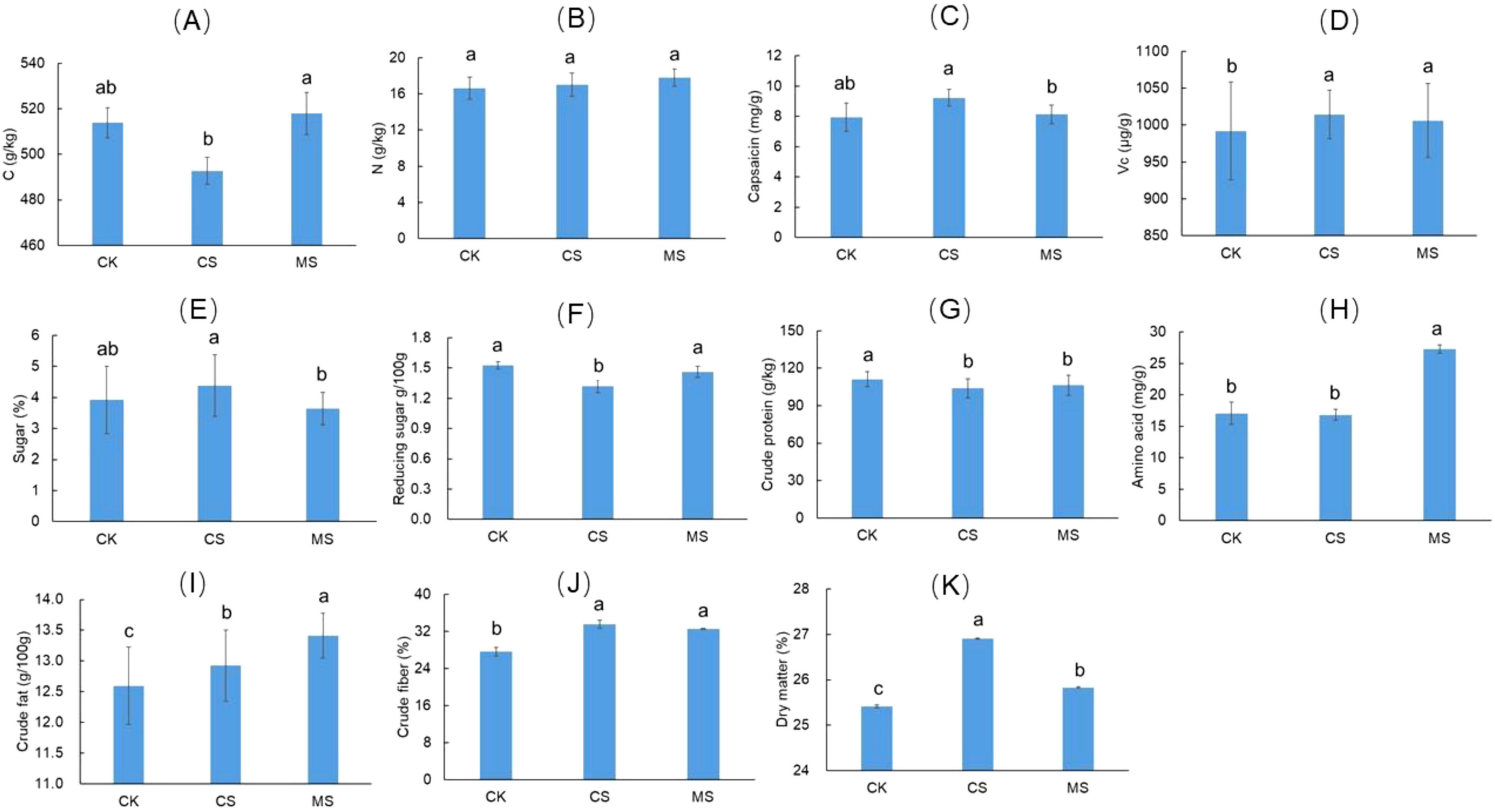
The nutritional quality of pepper under different treatments. **(A)** represents carbon content; **(B)** represents nitrogen content; **(C)** represents capsaicin content; **(D)** represents Vitamin C content; **(E)** represents total sugar content; **(F)** represents reducing sugar content; **(G)** represents crude protein content; **(H)** represents amino acid content; **(I)** represents crude fat content; **(J)** represents crude fiber content; **(K)** represents dry matter percentage. Different lowercase letters in each fighre indicate significant differences among treatments at the *P* < 0.05.

Comparative analysis of nutritional quality parameters in pepper fruits under distinct straw return treatments revealed significant differences across treatments, as demonstrated in **Figure 7** (*P*<0.05). Compared to CK, the CS treatment reduced pepper C content by 4.1%, while MS increased C content by 0.8%. Both CS and MS treatments enhanced N content by 2.4-7.2%, though without statistical significance. Capsaicin content increased by 16.5% (CS) and 2.5% (MS), whereas vitamin C (Vc) levels rose by 2.2% (CS) and 1.4% (MS) relative to CK. The CS treatment elevated total sugar content by 12.8%, contrasting with a 7.7% reduction under MS. Notably, CS decreased reducing sugar content by 13.3%. Crude protein content declined by 6.6% (CS) and 4.4% (MS). Amino acid content surged by 59.6% under MS versus CK. Both treatments increased crude fat (CS: +2.4%; MS: +6.3%), crude fiber (CS: +21.4%; MS: +17.8%), and dry matter content (CS: +5.9%; MS: +1.7%).

## 4 Discussions

### 4.1 Mechanism of straw returning on soil nutrients and microbial biomass

The results of this study demonstrated significant differential impacts of CS and MS treatments on soil properties (**Table 1**). CS exhibited a lower C/N ratio (24.3), with its cellulose-dominated structure facilitating rapid microbial decomposition, leading to short-term release of labile carbon sources and enhanced organic matter mineralization (Jin et al., 2020b). However, SOM content under CS treatment decreased compared to the control (12.7 g/kg vs. CK 13.8 g/kg), likely attributable to carbon pool depletion caused by accelerated cellulose degradation. This observation aligns with Yang et al. (2020), who reported transient declines in soil organic carbon during initial phases of corn straw incorporation due to preferential cellulose decomposition. In contrast, MS displayed superior lignin content (C/N ratio 39.2), where its recalcitrant aromatic architecture delayed decomposition kinetics while promoting humification processes (Huang et al., 2021b). Consequently, MS treatment significantly elevated SOM content (15.2 g/kg vs. CK 13.8 g/kg), consistent with Wang et al. (2021c) regarding lignin-rich straws enhancing long-term carbon sequestration.

Both straw treatments significantly enhanced soil TN and NH_4_^+^-N concentrations (**Table 1**). CS treatment released nitrogen through rapid decomposition of cellulose, but MS with high C/N ratio might trigger microbial N fixation (Wang et al., 2021b), resulting in a TN increase comparable to that of CS. As we all know, excessive accumulation of soil nitrate nitrogen enhances nitrogen availability, but weakens microbial mediated carbon nitrogen coupling efficiency by inhibiting actinomycete abundance and reducing enzyme activity (Wang et al., 2024; Zhang et al., 2024a). The lignin degrading microbial community regulates nitrogen storage buffer capacity through nitrate nitrogen chelation, maintaining microbial metabolic homeostasis (Chen et al., 2024a). It is worth noting that NO_3_^–^N content increased significantly under CS treatment (21.2 mg kg^-1^ vs. CK 6.8 mg kg^-1^), which might be attributed to cellulose promoting the activity of nitrifying bacteria (such as *Gammaproteobacteria* in *Proteobacteria*) (Yuan et al., 2023a). The decrease of NO_3_^–^N content to 2.0 mg kg^-1^ under MS treatment might be related to the inhibition of nitrification by the competitive utilization of N by microorganisms during lignin decomposition (Yang et al., 2020). In addition, MS treatment significantly increased the content of AK (438.5 mg kg^-1^ vs. CK 307.5 mg kg^-1^), which was consistent with the characteristics of mulberry rich in K (Qin, 2021).High concentrations of available potassium in soil could enhance aggregate stability (Zhang et al., 2023b). The complexation of potassium ions with humic acid could lead to the accumulation of recalcitrant carbon components (Li et al., 2024a). In summary, cellulose and lignin regulated carbon and nitrogen balance through differentiated decomposition pathways. The former promoted short-term mineralization but might lead to carbon loss, while the latter achieved carbon sequestration and nutrient release through slow decomposition (Raza et al., 2023a).

MBC and MBN were key indicators reflecting soil active nutrient pool and microbial metabolic status. The decrease in MBN could lead to a decrease in organic matter stability while MBN promoted the formation of humus and enhanced the synergy of microbial carbon and nitrogen metabolism (Yang et al., 2022; Zhang et al., 2023a; Chen et al., 2024b). The differences in soil MBC and MBN between MS and CS treatments were closely related to the chemical composition and C/N ratio of straw. The high lignin content of MS provided a stable carbon source for microorganisms and supported long-term community proliferation (Wu et al., 2020). Although the low C/N ratio of CS could rapidly release nitrogen, it might lead to a large consumption of nitrogen sources by microorganisms in the short term, resulting in a decrease in MBN (Vance et al., 1987). This was consistent with the study of Tan et al. (2021), which pointed out that high carbon and nitrogen were more conducive to maintaining the stability of MBN than straw (such as wheat straw). In addition, CS treatment significantly reduced CEC, which might be due to the fact that organic acids produced by cellulose decomposition reduced soil colloid stability (Raza et al., 2023b), which in turn affected the adsorption capacity of microorganisms to nutrients. In contrast, the effect of MS treatment on CEC was smaller, indicating that the destruction of lignin decomposition products on soil structure was weaker. The above results were consistent with the findings of Ninkuu et al. (2025) that lignin-derived phenolics could enhance the stability of soil aggregates by chelating metal ions. Therefore, lignin straw returning was more conducive to maintaining the long-term balance of MBC and MNB, while cellulose straw might indirectly affect microbial metabolism by changing soil physical and chemical properties.

### 4.2 Mechanism of straw returning on soil microbial community structure

*Proteobacteria* enhances soil nutrient availability by driving carbon and nitrogen cycling (Smith et al., 2022). *Actinobacteria* enhance microbial community stability and inhibit pathogen proliferation by degrading stubborn organic matter and secreting antibiotic substances (Wang et al., 2021a). *Acidobacterium* maintains microbial balance by regulating organic acid metabolism under low pH conditions, but some of its taxa may compete for nitrogen resources, leading to heterogeneity in fertility (Kim et al., 2023). *Pseudomonadota* was the dominant phylum, and its members (such as *Pseudomonas*) played a central role in the nitrogen cycle (**Figure 2**). CS treatment did not significantly change its abundance, but might promote NO_3_^–^N accumulation by increasing the activity of nitrifying bacteria (such as *Gammaproteobacteria*) (Yuan et al., 2023a). This mechanism was consistent with the study of Zou et al. (2022a) on nitrifying bacteria in the rhizosphere of pepper. The abundance of *Actinomycetota* increased in CS treatment, and its secreted lignin peroxidase and cellulase could synergistically degrade complex organic matter (Ninkuu et al., 2025), and the antibiotics produced might inhibit soil-borne pathogens (Ma et al., 2023). The abundance of *Acidobacteriota* decreased in MS treatment, which might change soil pH due to lignin decomposition products (such as phenols), while *Acidobacteriota* prefered acidic environment (Raza et al., 2023b). This result was consistent with the finding of Alami et al. (2021), that is, lignin derivatives could significantly reduce the relative abundance of Acidobacteria.

PCoA analysis showed that CS treatment significantly changed the microbial community structure (**Figure 3**), while MS treatment overlapped more with CK, indicating that cellulose straw had a stronger disturbance on community composition. LEfSe analysis further showed that actinomycetes (*Micrococcales*) were significantly enriched in CS treatment (**Figure 4**), a group known for secreting cellulases (Tan et al., 2021), and the increase in abundance might be related to increased demand for cellulose degradation. In contrast, MS treatment did not significantly change the proportion of dominant bacteria, but the Circos plot showed that the relative abundance of *Acidobacteriota* decreased, which might be related to the inhibition of the growth of *Acidobacteriaceae* by phenols produced by lignin decomposition (Alami et al., 2021). This finding was consistent with the report of Ma et al. (2023) that lignin derivatives (such as vanillic acid) could selectively inhibit the activity of *Acidobacteria*. In summary, straw types selectively regulated the abundance of functional bacteria by changing substrate availability and soil environment. Cellulose straw enriches cellulose-degrading bacteria (such as *actinomycetes*), while lignin straw inhibits specific bacteria (such as *Acidobacteria*) through allelopathy (Raza et al., 2023a).

The correlation analysis between bacterial and fungal functions and soil nutrients reveals the key pathways of microbial driven nutrient cycling. The positive correlation between SOC and MBN indicates that microorganisms convert organic carbon into active nitrogen pools through assimilation, promoting nitrogen availability (Liang et al., 2020). The synergistic enhancement of NO_3_^–^N by bacteria and fungi suggested that they dominated the nitrification process, while the negative correlation of NH_4_^+^-N might reflect the rapid conversion of ammonium to nitrate, or microbial preference for nitrate nitrogen absorption (Kuzyakov and Xu, 2023).

### 4.3 Response characteristics of pepper quality indicators to straw returning

An increase in capsaicin content can enhance antioxidant and anti-inflammatory activity (Zou et al., 2022b), thereby increasing the commercial value of seasonings. Although the increase in coarse fiber improves the texture of the product, it may reduce its palatability. The increase in amino acids and crude fat enhances nutritional function by providing essential amino acids and unsaturated fatty acids. The reduction of reducing sugars can weaken the perception of sweetness (Li et al., 2024b). Straw returning indirectly regulated pepper quality by improving soil nutrients and microbial activity (**Figure 7**). CS treatment significantly increased capsaicin content, which might be related to the increase of AK content to promote the synthesis of secondary metabolites (Zou et al., 2022a). MS treatment significantly increased amino acid content, because lignin slow-release nitrogen prolonged the plant absorption cycle (Huang et al., 2021b), which was consistent with the conclusion of Qin (2021) on mulberry returning to improve crop nitrogen use efficiency. In addition, CS treatment increased crude fiber and dry matter, which might be related to the increase of MBC to promote cell wall synthesis (Yuan et al., 2023a). However, CS treatment reduced the content of reducing sugar, which might inhibit the accumulation of sugar due to the imbalance of carbon-nitrogen ratio (Mou et al., 2020). In contrast, MS treatment increased crude fat and vitamin C, reflecting that lignin decomposition products (such as phenols) might protect plant cells through antioxidant effects (Yang et al., 2023).

Studies have shown that soil C/N synergistically affects crop quality by regulating carbon and nitrogen resource allocation. For example, a significant increase in soil C/N might be associated with enhanced carbon metabolism promoting the synthesis of secondary metabolites in capsaicin content (Zhao et al., 2021). Under high C/N conditions, the accumulation of crude fibers might be driven by carbon enrichment, which drived the biosynthesis of cell wall components such as cellulose (Ding et al., 2020). However, the total amount of amino acids increased with the decrease of C/N, reflecting active protein synthesis when nitrogen was sufficient, while crude fat showed a decreasing trend due to carbon allocation competition (Smith-Moore et al., 2018).

## 5 Conclusions

This study revealed that cellulose CS significantly increased nitrate nitrogen and available potassium content through rapid mineralization, directly driving the synthesis of capsaicin and crude fiber. However, its short-term mineralization process leaded to a decrease in soil organic matter and microbial nitrogen, indicating that carbon pool loss and nitrogen imbalance risks might limit long-term soil health. In contrast, the lignin MS significantly enhanced organic matter and microbial biomass carbon through lignin slow-release properties, and promoted the accumulation of amino acids and crude fat in chili peppers, highlighting its advantages in carbon sequestration and long-term nutrient supply. Based on the above results, the straw returning strategy in pepper cultivation in karst areas needs to balance short-term fertilizer efficiency and long-term ecological benefits. For nutrient poor soils, it is recommended to prioritize the use of CS treatment to rapidly increase the supply of available nutrients, but it needs to be combined with organic fertilizer or green manure rotation to alleviate carbon and nitrogen imbalance. For areas with severe degradation or organic matter deficiency, MS treatment can be used as a core measure to gradually increase SOM content and enhance soil water and fertilizer retention capacity through lignin slow-release properties. The selection of straw types for future research needs to be combined with soil health monitoring and the characteristics of soil erosion in karst areas.

## Data Availability

The original contributions presented in the study are included in the article/supplementary material. Further inquiries can be directed to the corresponding author.
